# Isolation and Identification of *Staphylococcus* Species Obtained from Healthy Companion Animals and Humans

**DOI:** 10.3390/vetsci9020079

**Published:** 2022-02-13

**Authors:** Pamela Thomson, Patricia García, Jorge Miles, David Isla, Camilo Yáñez, Rodrigo Santibáñez, Andrea Núñez, Carla Flores-Yáñez, Camila del Río, Françoise Cuadra

**Affiliations:** 1 Laboratorio de Microbiología Clínica y Microbioma, Escuela de Medicina Veterinaria, Facultad de Ciencias de la Vida, Universidad Andrés Bello, Santiago 8370134, Chile; kamidelrio@gmail.com (C.d.R.); francoise.cuadra@live.cl (F.C.); 2 Departamento de Laboratorios Clínicos, Facultad de Medicina, Pontificia Universidad Católica, Santiago 8940000, Chile; pgarciacan@uc.cl (P.G.); jmmiles@uc.cl (J.M.); david.isla@uc.cl (D.I.); ciyanez1@uc.cl (C.Y.); 3 Departamento de Ingeniería Química y Bioprocesos, Facultad de Ingeniería, Pontificia Universidad Católica, Santiago 8940000, Chile; rlsantibanez@uc.cl; 4 Facultad de Medicina Veterinaria y Agronomía, Universidad de las Américas, Santiago 7500975, Chile; andrean27@gmail.com; 5 Clínica Veterinaria Los Avellanos, Santiago 8380239, Chile; veterinarialosavellanos@gmail.com

**Keywords:** antimicrobial susceptibility testing, human, dogs, cats, *Staphylococcus* spp., resistance

## Abstract

The close contact between people and their pets has generated the exchange of skin microbiota, accompanied by bacteria that present resistance to antibiotics. *Staphylococcus* spp., opportunistic pathogens present in the skin and mucosa of mammals, have had their importance recognized in human and veterinary medicine. The objectives of this study were to identify *Staphylococcus* spp. present in isolates from the nostrils of healthy humans, dogs and cats as well as to determine their phenotype of resistance to methicillin. Strain identification was performed by MALDI-TOF mass spectrometry and antimicrobial susceptibility was determined using a disk diffusion assay for 12 antibiotics. Sixty humans (veterinary and technicians), sixty dogs and sixty cats were sampled; of them, 61.6%, 56.6% and 46.6%, respectively, carried *Staphylococcus* spp. in their nostrils, and only two people carried two different species of *Staphylococcus* in the only anatomical site sampled. A methicillin-resistant phenotype was present in 48.7% of the humans, 26.5% of the dogs and 57.1% of the cats, and sampled. These results demonstrate the presence of *Staphylococcus* spp. strains resistant to methicillin in personnel who work in contact with animals, as well as in dogs and cats that entered the same hospital or veterinary clinic, which alerts us to the potential transfer of these strains to or between people, dogs and/or cats.

## 1. Introduction

The genus *Staphylococcus* is composed of Gram-positive and facultative anaerobic bacteria present in cutaneous and mucous membrane microbiota of mammals and birds [1,2]. The genus includes clinically relevant opportunistic pathogens in both human and veterinary medicine [3,4,5,6]. The species belonging to this genus have traditionally been grouped and differentiated according to the production of the enzyme coagulase, capable of converting fibrinogen into fibrin, a characteristic that is easily detectable in the laboratory and allows for a practical classification [7]. In general, coagulase-positive staphylococci (CoPS), such as *S. aureus*, *S. intermedius* and *S. pseudointermedius*, among others, are usually pathogenic, even though in some cases they can cause asymptomatic colonization in healthy individuals, whereas coagulase-negative staphylococci (CoNS) [8], represented by a larger group of species, have been associated with opportunistic infections [3,4,9,10,11,12,13].

For decades, *S. aureus* has been considered the most important pathogen of the genus [14,15,16,17]. In people, it can be found in community settings [18] or hospital premises, constituting an important source of infections associated with healthcare [19]. The bacterium can produce infections in humans associated with skin and soft tissue, pneumonia, septicemia and osteomyelitis [19], which have also been reported in animals [20]. However, *S. pseudointermedius* nevertheless is a common cause of skin and soft tissue infections in dogs, cats and humans [5,6,21]. In recent years, CoNS species, such as *S. epidermidis* [22,23,24], *S. haemolyticus* [25] and *S. lugdunensis* [26], have also been associated with opportunistic infections in humans [12,23,27,28,29]. The recognition of some CoNS as pathogens in veterinary medicine has emerged with *S. epidermidis* and some subspecies of *S. schleiferi* causing skin and ear infections in dogs [30,31,32,33], as well as *S. felis* related to lower urinary tract disease, eye infections and otitis in cats [34,35].

Pathogen transmission between species is recognized as being of clinical relevance and zoonotic. The transmission of commensal *Staphylococcus* spp., including those resistant to methicillin and other antibiotics, has been recognized from animals to humans and vice versa [35], particularly among domestic animals and their owners [32,36,37,38,39,40,41,42,43,44]. Importantly, the transmission also occurs among staff working in veterinary hospitals and their patients, as well as among patients who are in the same hospital [5,45,46,47,48,49,50]. The objective of this study was to isolate and identify *Staphylococcus* spp. obtained from healthy humans, dogs and cats.

## 2. Materials and Methods

### 2.1. Ethical Approval

This study was approved by the Bioethics Committee of the Faculty of Life Sciences, Universidad Andrés Bello (Approval Certificate #019/2020), and was carried out in a veterinary hospital and a veterinary clinic, located in Colina and Independencia, respectively, Santiago, Metropolitan Region, Chile (S 33°27′24.98″ O 70°38′53.77″). Samples were collected between October 2020 and March 2021.

### 2.2. Subjects and Inclusion Criteria

Sixty healthy adult dogs and sixty healthy adult cats of any breed and sex were included, who attended, with their owners, the hospital or veterinary clinic to comply with their vaccination schedule. Similarly, 60 people were sampled during the same period. Sampled people were veterinary doctors and technicians who worked regularly at or visited the same hospital or clinic. Included subjects or enrolled pets were under no antibiotic treatment for at least 3 months before obtaining the sample.

### 2.3. Isolation and Identification

Each sample was obtained with prior authorization by means of informed consent. For each sampled subject, the use of a face shield, mask and sterile gloves was taken into consideration, materials which were discarded between each participant. In humans, a single swab was inserted by a maximum of 1 cm, rotated in each nostril and rubbed with support on the septum. In animals the same procedure was carried out, considering that in small breeds and cats the swab was introduced by a maximum of 0.5 cm and employed Stuart Transport media (Linsan, Santiago, Chile). Each swab was seeded on mannitol salt agar (Becton Dickinson, Heidelberg, Germany) and incubated at 37 °C for 24 h; a semi-quantitative evaluation was made of the different morphotypes grown on mannitol salt agar, such that those that showed abundant growth in the second quadrant of the clock sowing were selected. Gram- and catalase-positive morphotypes were isolated on blood agar (Linsan, Santiago, Chile); additionally, they were identified using matrix-assisted laser desorption/ionization time-of-flight (MALDI-TOF) mass spectrometry analysis (MALDI Biotyper, Bruker, Billerica, MA, USA) following the manufacturer’s instructions and as described previously [51,52].

### 2.4. Antimicrobial Susceptibility Testing

All isolates confirmed as *Staphylococcus* were tested against a panel of 12 antibiotics using the disk diffusion Kirby–Bauer method following CLSI guidelines in the M100 and VET01S documents [53,54]. The tested antibiotics included cefoxitin (FOX, 30 µg), oxacillin (OX, 1 µg), imipenem (IPM, 10 μg), ciprofloxacin (CIP, 5 μg), vancomycin (VA, 30 μg), doxycycline (DO, 30 μg), erythromycin (E, 15 μg), amikacin (AMK, 30 μg), gentamicin (GEN, 10 μg), trimethoprim/sulfamethoxazole (SXT, 1.25/23.75 μg), amoxicillin/clavulanic acid (AMC, 30 μg) and clindamycin (DA, 2 μg), all of which were supplied by OXOID (Hampshire, UK). Methicillin-resistant phenotype in strains of human origin were evaluated using a FOX disc for all species. However, in cats and dogs, OX for *S. pseudointermedius* and CoNS as well as FOX for *S. aureus* were used.

*S. aureus* ATCC 25923 was included as a reference strain. Bacterial isolates resistant to three or more antimicrobial classes were cataloged as multidrug-resistant (MDR) following previously standardized criteria [55].

### 2.5. Data Analysis

Data were analyzed and plots were made utilizing python3 software and the pandas package, release 1.3.4; upset plots [56] were made by employing the UpSetPlot package (https://github.com/jnothman/UpSetPlot (accessed on 27 October 2021), release 0.6.0.

## 3. Results

### 3.1. Study Population

The group of 60 people that were sampled consisted of 35 women and 25 men with an average age of 30 years (range of 21 to 44 years), who carried out different activities within the clinic or hospital. Activities recorded were surgeon, surgeon assistant, animal care and treatment, management of hospitalized, medical consultation, wound treatment, student veterinary, nurse, animal rehabilitation, feline care and analysis of animal samples, among others.

A total of 120 pets (60 dogs and 60 cats) were sampled from a veterinary hospital and clinic in Santiago, Chile. Half of the dogs were crossbreeds (30), while the others were German Shepherds (6), Poodles (4), Schnauzers (3), Cocker Spaniels (3), Yorkshires (3), Beagles (2), Labradors (2), Chihuahuas (2), a Dachshund (1), a Maltese (1), a Saint Bernard (1), a Pug (1) and an Akita (1). Their average age was 4.4 years (range of 2 to 9 years). The cats had an average of 4.3 years (range of 2 to 8 years), and most of them were domestic short hairs (41), domestic long hairs (18) and a Siamese (1).

### 3.2. Detection of Staphylococcus spp.

Of the total number of humans, 61.6% of the subjects sampled (37 of 60) carried a *Staphylococcus* spp. in their nostrils; only two people carried two different species of *Staphylococcus* spp. in the only anatomical site sampled, meaning that a total of 39 isolated strains were obtained. Of the dogs and cats, 56.6% (34 of 60) and 46.6% (28 of 60) were carriers of *Staphylococcus* spp., respectively.

**Figure 1:**
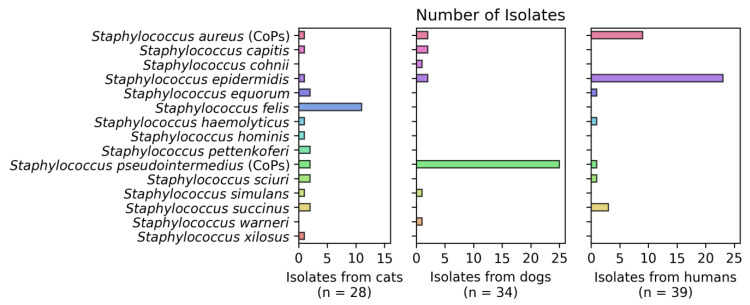
Number of *Staphylococcus* isolates per group. In total, 15 different species from the *Staphylococcus* genus were isolated from the studied subjects. Considering the frequency, 28 *Staphylococcus* spp. were isolated from the 60 healthy adult cats, 34 *Staphylococcus* spp. were isolated from the 60 healthy adult dogs and 39 *Staphylococcus* spp. were isolated from the 60 healthy adult human participants of the study.

A total of 13 species of CoNS were identified, the most prevalent being *S. epidermidis* (26.1%), *S. felis* (11.1%), *S. succinus* (4.8%), *S. sciuri* (2.9%) and *S. equorum* (2.9%), distributed among the humans and animals sampled (Figure 1).

In humans, most of the isolates corresponded to CoNS represented by *S. epidermidis*, with 58.9% being of this type (23 of 39 isolates). This also occurred in cats, where 39.3% of the isolates were *S. felis* (11 of 28). On the contrary, in dogs two species of CoPS were identified, represented by *S. pseudointermedius* (25 of 34) and *S. aureus* (two of thirty-four) (Figure 1).

### 3.3. Resistance Phenotype

**Figure 2:**
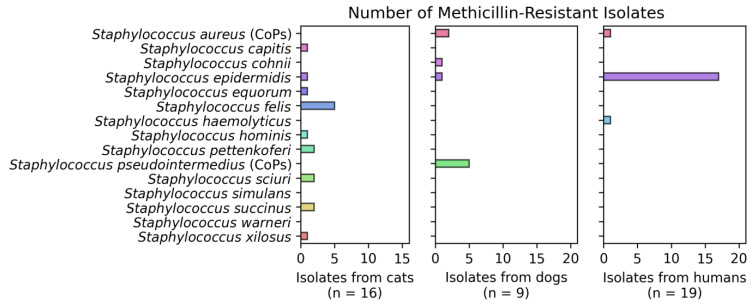
Number of methicillin-resistant isolates per group (cats, dogs and humans).

Of all the isolates obtained from humans, 48.7% (19 of 39) showed a methicillin-resistant phenotype, mainly in isolates of *S. epidermidis* (17), *S. aureus* (1) and *S. haemolyticus* (1). The strains isolated from dogs showed 26.5% resistance, where *S. pseudointermedius* (5) was the predominant species, followed by *S. aureus* (2), *S. cohnii* (1) and *S. epidermidis* (1). Likewise, 57.1% of the isolates from felines showed this phenotype in the species *S. felis* (5), followed by *S. sciuri* (2), *S. pettenkoferi* (2), *S. succinus* (2), *S. capitis* (1), *S. xilosus* (1), *S. epidermidis* (1), *S. hominis* and *S. equorum* (1) (Figure 2).

**Figure 3:**
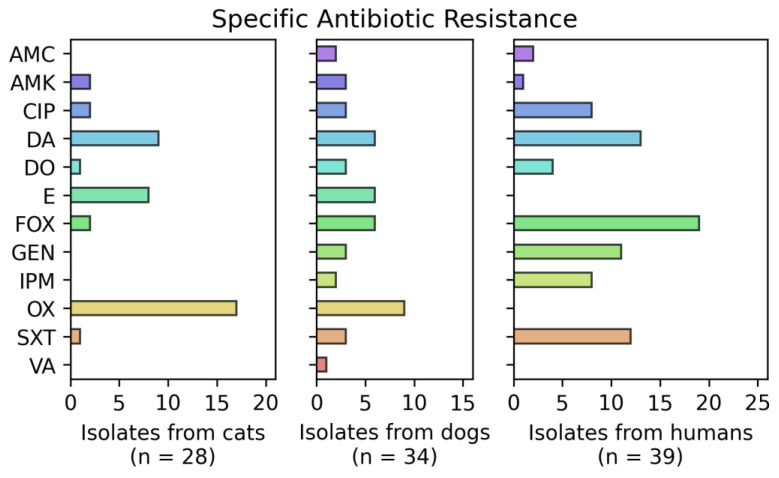
Number of resistant isolates obtained from dogs, cats and humans. All isolates were tested for antimicrobial susceptibility employing the disk diffusion method following CLSI guidelines. Colors represent the total number of *Staphylococcus* isolates that showed resistance to cefoxitin (FOX, 30 µg), oxacillin (OX, 1 µg), imipenem (IPM, 10 μg), ciprofloxacin (CIP, 5 μg), vancomycin (VA, 30 μg), doxycycline (DO, 30 μg), erythromycin (E, 15 μg), amikacin (AMK, 30 μg), gentamicin (GEN, 10 μg), trimethoprim/sulfamethoxazole (SXT, 1.25/23.75 μg), amoxicillin/clavulanic acid (AMC, 30 μg) and clindamycin (DA, 2 μg).

**Figure 4:**
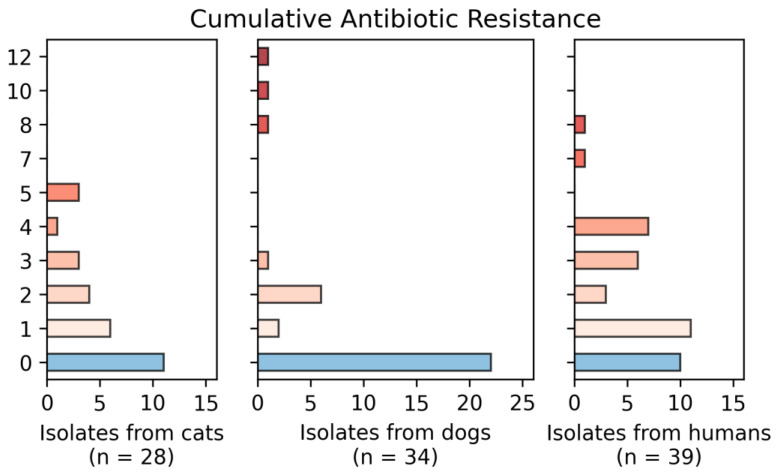
Cumulative resistance of the number of isolates obtained from dogs, cats and humans against the antibiotics used. In total, 11 isolates from cats, 22 from dogs and 2 from human participants showed resistance to zero antimicrobials. Two isolates showed resistance to nine, and one isolate showed resistance to 10, 11 and 12 antimicrobials.

**Figure 5:**
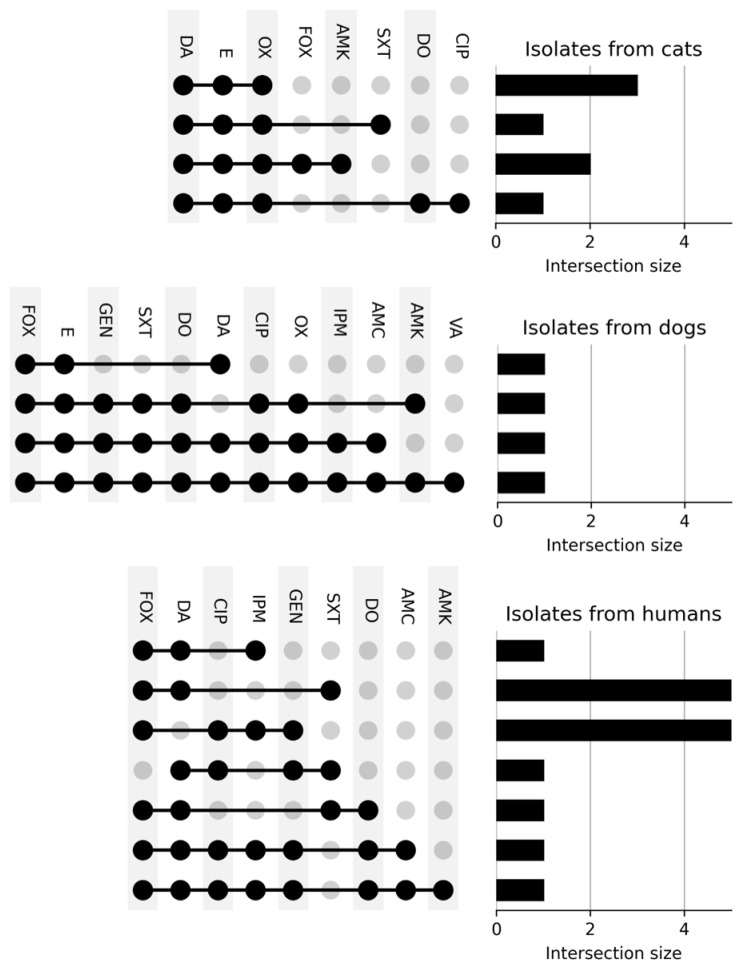
Specific multidrug resistance of isolates. In total, seven isolates from cats, four isolates from dogs, and fifteen isolates from human participants showed resistance to three or more antibiotics. All isolates from cats showed resistance to OX, E and DA. Isolates from dogs showed elevated multidrug resistance, with all isolates being resistant to E as well as DA and three of them also showing resistance to GEN, SXT, DO, CIP and OX. Finally, most of the isolates from human participants showed resistance to FOX and DA.

Figure 3, Figure 4 and Figure 5 show the number of isolates obtained from dogs, cats and humans that were resistant against the different antibiotics tested. It is noted that 11 isolates from cats, 22 from dogs and 2 from human participants showed resistance to zero antimicrobials; seven isolates from cats, four isolates from dogs and fifteen isolates from humans showed resistance to three or more antibiotics. All cat isolates showed resistance to OX, E and DA. Isolates from dogs showed high multidrug resistance, with all of them resistant to E and DA and three of them also resistant to GEN, SXT, DO, CIP and OX. Of all the isolates there was a pan-resistant *S. pseudointermedius* strain, isolated from a dog.

## 4. Discussion

This is the first study carried out in Santiago de Chile that informs on the diversity of CoPS and CoNS species present in the nostrils of healthy humans, dogs and cats, and that also reports the resistance of those obtained against 12 antibiotics.

In general, the frequency of isolates was similar in the three groups sampled; however, the presence of CoPS or CoNS differs between them and is consistent with previous studies; for example, one carried out in Trinidad and Tobago shows a general carriage of *Staphylococcus* in 53.4% of pets and 46.6% of their owners, indicating that these strains can act as a reservoir of resistance genes between dogs and humans [46]. Other reports have evaluated the carriage of CoPS, mainly *S. aureus* and *S. pseudointermedius* in animals, with quantities that fluctuate between 8.7 and 43.8% [6,21,28,40]. Likewise, some authors have focused on the detection of CoNS obtained from healthy animals, finding a carriage that varies between 12.8 and 28% [12,21,22,30].

In Latin America there is little information about the carriage and resistance levels of *Staphylococcus* spp. in companion animals; a retrospective study conducted in Argentina analyzed a total of 23,922 isolates recovered from clinical samples of dogs and cats between 2011 and 2017, of which 30.8% corresponded to three species of *Staphylococcus* spp., with *S. pseudointermedius* being the most frequent [57] and therefore in agreement with what was reported in this work.

Different species implicated in human and animal infections were isolated, such as *S. aureus*, *S. epidermidis* and *S. pseudointermedius* [22,23,24]. Additionally, other pathogens considered to be emerging, such as *S. sciuri, S. simulans, S. haemolyticus, S. schleiferi* and *S. lugdunensis*, were isolated [12,23,27,30,31,32]. In humans, most of the isolates corresponded to CoNS represented by *S. epidermidis*, considered to be the most abundant species that lives on skin [55]. In recent years this species has been associated with infections in humans, dogs and cats; it has also been related to resistance to methicillin, which makes some infections difficult to treat [12,22,25,31]. In cats, *S. felis* (CoNS) is a species that has been reported as the most frequent [21,28,33,34,35], and has been associated with lower urinary tract disease, eye infections and otitis in these pets [34,35].

Of CoPS species, *S. pseudointermedius* was the most isolated in this study mainly in dogs, a worldwide concordant finding, reflecting the adaptation of this *Staphylococcus* to dogs as the major host species [28,40,43,46,48]. Furthermore, it has acquired great importance in these patients as it is one of the main species causing deep pyoderma [33,58]. The isolation of this species in felines differs enormously and depends on the anatomical site sampled: cases in the nostrils of healthy animals are lower compared to skin scraping [33].

A significant number of isolates were found to have a methicillin-resistant phenotype present, specifically in 26.5% of the dogs, 57.1% of the cats and 48.7% of the humans sampled. These figures are higher compared with previous studies carried out in Africa [21], Spain [58] and Australia; additionally, the latter indicates the absence of the phenotype in felines [33].

*S. aureus* and *S. pseudointermedius* were isolated from humans and dogs. One isolate of *S. aureus* from humans and two isolates from dogs were methicillin-resistant. However, only *S. pseudointermedius* isolates from dogs showed resistance to methicillin. Regarding this, previous studies have detected variable numbers of resistance in pets from 2.6% [33] to 27.4% [6], while others mention 13.3% and 15.1% in CoPS and CoNS, respectively [45]. This scenario shows us limited therapeutic options to treat infections generated by methicillin-resistant *Staphylococcus* either in humans or animals; in such cases the options are limited to drugs such as vancomycin, oxazolidinones, daptomycin, tigecycline and novel cephalosporins [59,60].

Interestingly, Rossi et al. show a dynamic of horizontal transfer of antimicrobial resistance genes from CoNS to other *Staphylococcus* species [22], while other authors point to *S. sciuri* (a colonizer found in dogs) as the source of the mecA gene present in *S. aureus* [61]. Regarding the risk factors that affect the carrying of a methicillin-resistant genotype, hospitalization, previous bacterial infections and the density of the human population have been reported, indicating a positive association between these variables [28].

Since, in 2017, the WHO classified those strains of *S. aureus* resistant to methicillin and vancomycin as “high priority” [53], in this study we obtained one isolate with this type of resistance corresponding to *S. pseudointermedius* obtained from a dog, an antecedent that we must consider since these microorganisms can transmit resistance genes to other species. On the other hand, of all the strains obtained 20 of them were MDR. Previously, it has been reported that 55% of the isolates of *S. aureus* and *S. pseudointermedius* obtained from pets were MDR [6], while other authors have obtained these results in 100% of the strains [5].

Interestingly, in people who carry *Staphylococcus* spp., resistance figures increase when they have been in direct contact with pets [39,42,58] that have been previously treated with antibiotics, as well as in those who work in hospitals or veterinary clinics [5], [32] expressing clonal lineages similar to those identified in humans and other animals [36,43,46,48]. Other authors report a prevalence of methicillin-resistant *S. aureus* between 0.2 and 15.3% among medical students [49] a figure that increases to 79% when the wardrobe, mainly long-sleeved gowns, has been sampled [47].

Considering that pets are a probable source of transmission of these agents, it is important to have trained professionals to supervise cleaning and disinfection protocols within a hospital or clinic, and on the other hand have detection, classification and isolation systems for high-risk patients in addition to contact tracing to prevent possible outbreaks by medical personnel.

## Data Availability

Not applicable.
